# Reliability and Validity of the Defeat Scale among Internal Migrant Workers in China: Decadence and Low Sense of Achievement

**DOI:** 10.3390/healthcare11060781

**Published:** 2023-03-07

**Authors:** Shangbin Liu, Yingjie Chen, Yaqi Chen, Tian Hu, Zixin Wang, Rongxi Wang, Fan Hu, Chen Xu, Xiaoyue Yu, Yujie Liu, Hui Chen, Danni Xia, Huachun Zou, Kechun Zhang, Bolin Cao, Meili Shang, Ying Wang

**Affiliations:** 1 School of Public Health, Shanghai Jiao Tong University School of Medicine, Shanghai 200025, China; 2 Shenzhen Longhua District Center for Disease Control and Prevention, Shenzhen 518109, China; 3 JC School of Public Health and Primary Care, Faculty of Medicine, The Chinese University of Hong Kong, Hong Kong 999077, China; 4 School of Public Health (Shenzhen), Sun Yat-Sen University, Shenzhen 528406, China; 5 Kirby Institute, University of New South Wales, Sydney 4385, Australia; 6 School of Media and Communication, Shenzhen University, Shenzhen 518061, China; 7 Sanlin Community Health Service Center, No. 375, Sanlin Road, Shanghai 200124, China

**Keywords:** defeat scale (DS), psychometric properties, internal migrant workers, reliability, validity

## Abstract

Introduction: Internal migrant workers have a great chance to experience defeat due to their low social status and economic situation. It has been reported that defeat might play a prospective role in predicting depression and anxiety; however, defeat is rarely explored among internal migrant workers due to the lack of appropriate measurement scales. The defeat scale (DS) can measure the feeling of defeat, social hierarchy reduction, and loss in social struggle. But its reliability and validity among internal migrant workers have not been reported. This study aimed to verify the content validity and structural validity of the DS among internal migrant workers in China and to explore its correlations with anxiety and depression. Methods: 1805 internal migrant workers (IMWs) were recruited by stratified multistage sampling from 16 factories in Shenzhen, China. The content validity index (CVI) was used to assess content validity. Cronbach’s coefficient alpha of each factor and the total scale were calculated to assess the reliability of DS. The scree test was used to determine the number of factors. Convergent validity and discriminant validity were estimated by calculating the average variance extracted and composite reliability. Logistic regression was performed to explore the effects of DS scores on anxiety and depression. Results: Mean score of DS among IMWs was 18.42 ± 9.40. There were 606 (33.6%) IMWs who were considered to have depression symptoms, and 524 (29.0%) IMWs were considered to have anxiety symptoms. A two-factor model was obtained and fitted well (CFI = 0.956, GFI = 0.932, IFI = 0.956, RMSEA = 0.068, SRMR = 0.052). Cronbach’s alpha reliability coefficient for the DS was 0.92. Logistic regression showed that DS scores were positively associated with anxiety and depression among IMWs. Conclusions: DS performed well among IMWs on content validity and structural validity, and it was suitable as a measurement instrument to assess defeat among this population. Defeat was positively associated with anxiety and depression and might play an important role in the mental health of IMWs.

## 1. Introduction

Since the 1980s, China has experienced rapid urbanization accompanied by massive migration from rural to urban areas. Internal migrant workers (IMWs) are registered in rural areas but work in big cities that are not part of the registered area [1]. Some of them go out to work only during the slack season, while some are permanently employed in cities [2]. In April 2021, data released by the National Bureau of Statistics showed that there were 285.6 million IMWs in China, accounting for 20% of the total population [3,4]. The living environment of IMWs in the city is not friendly, and the resulting various psychological problems attract much attention. However, few studies focus on their sense of defeat.

Defeat was included in the framework of social hierarchy theory [5]. It refers to the subjective feeling of defeat in a struggle and powerlessness, which is associated with the loss of social status and the failure of personal goals. Some studies reported that defeat was important in predicting the development of psychopathological syndromes, such as anxiety and depression [6]. Gilbert proposed three major categories of events that can trigger the sense of defeat: (1) failure to obtain or maintain access to social or material resources, (2) social slights or attacks from others, (3) “attacks” launched from within the individual, for example, in the form of excessive self-criticism, unfavorable social comparison processes, and unattainable goals [7]. IMWs might experience all these events and feel defeated in an unfriendly urban living environment.

IMWs are believed to be unfairly treated in urban life, characterized primarily by marginalization in multiple aspects of urban society, including housing, employment, education, health, and other services [8,9]. They are also deprived of some social welfare because of China’s household registration system [10]. These can be considered the first type of events that can induce a sense of defeat.

Some studies found that IMWs were more likely to experience discrimination, stigmatization, and spatial exclusion in their host cities [11,12,13]. Becker’s taste-based model of discrimination posits that discrimination is fundamentally an irrational prejudice that is indulged by employers [14]. It is quite possible that some urban employers consider IMWs to be inferior and refuse to believe that it is possible for IMWs to have the same productive characteristics as the majority of the urban population. IMWs live in cities with their rural lifestyles, behaviors, and dialects, but these are considered rude and uncivilized. In addition, the majority of IMWs have low levels of education and less urban working experience, which makes them receive less reward for the same work compared with their urban colleagues [15]. This can be considered the second type of event that can induce a sense of defeat.

According to previous studies, IMWs often work in dirty, dangerous, demanding, long-term and low-paying jobs in cities that other local citizens do not want to do [16,17,18,19,20], and they often live in crowded, chaotic, and unhealthy communities [21]. Such work and life environments might lead them to consider themselves losers. In addition, they may also blame themselves for their children’s inability to successfully receive education in big cities [22]. Another point worth noting is that IMWs work in cities for long periods of time, and the guilt of not being able to take care of their families adds to their frustration and stress [23]. This can be considered the third type of event that can induce a sense of defeat.

There is a great chance that IMWs might feel defeated due to the trigger events. It is necessary to pay attention to their sense of defeat. However, defeat is rarely explored among IMWs. One of the possible reasons could be the lack of suitable measurement scales. The defeat scale (DS) can measure the feeling of failure, social hierarchy reduction, and loss in social struggle [5]. It has been reported to be effective in multiple populations, such as students, prison inmates, and depressed people in the UK, Internet users in Germany, and university students in Turkey [5,24,25]. But it has not been validated in IMWs. Therefore, the purpose of this study is to apply the DS to IMWs and to report results on the reliability and validity tests. In addition, we will explore its correlations with anxiety and depression.

## 2. Methods

### 2.1. Sample Size

This study was designed to test the reliability and validity of the DS in IMWs. The sample size required in scale method studies is at least 10 times the number of scale items [26,27]. For this study, the DS was composed of 16 items, thus requiring a minimum sample size of 160 participants. This study recruited a sample of 1805 migrant workers, which was considered adequate for the purposes of this study.

### 2.2. Sampling

The city of Shenzhen boasts a resident population of 17.68 million, making it the largest internal migrant city in China. This large population of internal migrants is predominantly comprised of workers employed in factories, making Shenzhen a suitable location for sampling in studies related to this demographic. According to the report of Shenzhen Statistical Yearbook, 9.2%, 48.4%, 3.7%, 0.8%, 0.6%, 4.3%, and 0.4% of the industrial manufacturing workers in Longhua District engaged in the machinery processing industry, electronic device manufacturing industry, printing and dyeing industry, chemical material industry, melting industry, and garment industry [28]. Based on the size of factories, 16 factories, including 4 machinery processing factories, 3 electronic device manufacturing factories, 3 printing and dyeing factories, 2 chemical material factories, 1 melting factory, 1 garment factory, 1 food and beverage manufacturing factory, and 1 other factory were randomly selected. Then three to four workshops were then randomly selected from each factory. Eligible participants were (1) aged 18 or above and (2) working full-time in local factories. All workers who met the inclusion criteria were invited to participate in a survey conducted by the Center for Disease Control and Prevention, Longhua District.

### 2.3. Data Collection

Eligible participants were invited to pay a visit to the Longhua District Center for Disease Control and Prevention. Trained fieldworkers briefed participants about the study and confirmed their eligibility to join the study. They were anonymous in this study and had the right to withdraw at any time without any consequences. Written informed consent was obtained from 2700 workers in the selected workshops, and 2023 workers completed a self-administered questionnaire. 218 cases were eliminated because they were not IMWs, and finally, we got 1805 samples of IMWs. The study was approved by the Ethics Committee of the School of Public Health, Sun Yat-Sen University (2019/3) and conducted under the guidelines of the Declaration of Helsinki.

### 2.4. Measurements

#### 2.4.1. Defeat Scale

The DS, developed by Gilbert et al. in 1998, is a one-factor scale consisting of 16 items that assess individuals’ perceptions of losing rank position and failed struggle during the past seven days. The Chinese version of the DS still retains 16 items, but a two-factor structure was reported in some studies [29,30]. Items of DS are Likert-scaled and include options between zero (rarely) to four (always). Among 16 items, 3 items were positively worded (e.g., “I feel that I am a successful person.”) and were scored in the opposite direction. The total score of DS ranges from 0 to 64, and the higher the score is, the stronger the sense of defeat is [5].

#### 2.4.2. Generalized Anxiety Disorder-7

The GAD-7 is a self-rated screening tool for the rapid detection of possible anxiety disorders. It consists of seven questions and is rated on a 4-point Likert scale [31]. A cutoff value of ≥5 was used for diagnosing anxiety. It has been validated in patients from China, Iran, and Ecuador in Chinese, and Spanish, respectively [32,33,34].

#### 2.4.3. Revised Version of the Center for Epidemiologic Studies Depression Scale

A revised version of the Center for Epidemiologic Studies Depression Scale (CES-D-10) was obtained by reducing 20 items from the original version to 10 items. CES-D-10 has demonstrated strong psychometric properties, indicated by good reliability and construct validity [35]. A score of 10 is recommended as the cutoff value of CES-D-10 [35] and the Chinese version of the CES-D-10 has also been widely used by various groups of people in China [28,36,37].

### 2.5. Statistical Methods

#### 2.5.1. Content Validity

Content validity reflects the degree to which items of a scale instrument are relevant to and representative of the targeted construct for a particular assessment purpose. The content validity index (CVI) is the most widely used method to quantify the content validity of a scale [38]. It can be calculated for each item (I-CVI) and for the content validity index of the entire instrument (Average—CVI).

The CVI is based on expert ratings of each item based on the content relevance or representativeness of the scale, usually on a 4-point Likert scale from 1 (not relevant or not representative) to 4 (extremely relevant or representative). For each item, the I-CVI can be calculated by counting the number of experts who rated the item as 3 or 4 and dividing that number by the total number of experts, that is, the proportion of agreement about the content validity of an item [39]. I-CVI ≥ 0.8 was considered to be acceptable [40]. Average-CVI (Ave-CVI) is defined as “the average proportion of items rated as 3 or 4 (valid) across the various experts” [41]. It is used to assess the overall content validity index of an instrument, and 0.8 is the lower limit for Ave-CVI to be acceptable [42]. In this study, 8 psychology experts participated in the content validity assessment of DS.

#### 2.5.2. Structural Validity Test by Factor Analysis

Factor analysis is divided into two parts, namely exploratory factor analysis (EFA) and confirmatory factor analysis (CFA) [43]. EFA is used to explore the main variables to create a model from a relatively large set of latent dimensions usually represented by a set of items [44,45,46,47]. CFA, as a form of structural equation modeling (SEM), is applied to test the proposed model by researchers [43]. In this study, the database (n = 1805) was randomly divided into group 1 (n = 856) and group 2 (n = 949), and EFA was performed first and CFA later.

##### Kaiser-Meyer-Olkin and Bartlett’s Test

Before conducting the factor analysis, the adequacy of the sample and the suitability of the data for factor analysis should be tested. The sampling adequacy can be assessed by examining the Kaiser-Meyer-Olkin (KMO) [43,48]. The results range from 0 to 1, and above 0.70 is considered sufficient for factor analysis. Bartlett’s test of Sphericity provides a chi-square output [48]. It indicates the matrix of items is not an identity matrix. The results should be significant (*p* < 0.05) for factor analysis [49,50].

##### Screen Test

The scree test is the best choice for determining the number of factors [43,51]. The scree test plot consists of eigenvalues and factors, and it is reliable when the sample size is at least 200. The scree test can separate the important or major factors from the minor or insignificant factors [52]. In the scree test plot, the number of data points above the break (not including the point at which the break occurs) is the number of factors to retain.

##### Confirmatory Factor Analysis

Based upon the exploratory model, confirmatory factor analysis (CFA) was executed by the maximum likelihood (ML) algorithm in t group 2 (n = 949). Goodness-of-fit index (GFI, expected to be >0.9), comparative fit index (CFI, expected to be >0.9), incremental fit index (IFI, expected to be >0.9), normed fit index (NFI, expected to be >0.9), Tucker-Lewis Index (TLI, expected to be >0.9), Chi-square to degrees of freedom ratio (CMIN/DF, expected to be <5), root mean square error of approximation (RMSEA, expected to be <0.08) and standardized root mean square residual (RMR, expected to be <0.08) were utilized to assess the goodness of fit of the model [53,54].

#### 2.5.3. Reliability Test

Reliability is broadly defined in the population as the ratio of the variance of true scores to the variance of observed scores, while Cronbach’s coefficient alpha is a statistic for assessing scale reliability based on internal consistency [55]. Cronbach’s coefficient alpha of each factor and the total scale were calculated. Cronbach’s coefficient alpha ≥ 0.7 represents a good correlation, and alpha < 0.5 represents an unacceptable correlation [48,56].

#### 2.5.4. Validity Test

##### Convergent Validity

Convergent validity was used to assess the degree of interrelationship for the items of each factor, and it was estimated by calculating the average variance extracted (AVE) and composite reliability (CR) [48,57]:

(1) $$AVE=\frac{\sum_{n=i}^{n}\lambda^{2}}{n},$$

(2) $$CR=\frac{{\left(\sum_{i=1}^{n}\lambda_{i}\right)}^{2}}{\left(\sum_{i=1}^{n}\lambda_{i}\right)+\left(\sum_{c=1}^{n}\delta_{i}\right)}.$$

n = Number of items for each factor; i = c = 1,2…n; λ = factor loadings; δ = item error).

Estimates of AVE ≥ 0.50 and CR ≥ 0.70 were considered to indicate a strong interrelationship of items [50,54]. Items with convergent validity factors have a high degree of similarity, measuring similar content [58,59].

##### Discriminant Validity

The discriminant validity assesses whether the items that reflect a factor are not strongly correlated with another factor [54]. AVE and Correlation coefficient between factors can help us determine the discriminant validity between factors. If the square root of the AVE of any two factors is greater than their correlation coefficients, then the two factors are considered to have discriminant validity [57,60]. The factors with discriminant validity differ in what they measure.

#### 2.5.5. Logistic Regression

IMWs with GAD-7 scores greater than 4 were considered to be suffering from anxiety, and IMWs with CES-D-10 scores greater than 10 were considered to be suffering from depression. Logistic regression was performed to explore the effect of DS scores on anxiety and depression.

## 3. Results

### 3.1. Descriptive Data

The average age of the participants was 32.0 (27.0–37.0) years old. Most IMWs (53.6%) were married, and 1214 (67.3%) were male. More than 80% of IMWs were of Han nationality. Most of the IMWs’ monthly income was 464.00–773.18$. The mean score of DS among IMWs was 18.42 ± 9.40. This was higher than students (17.2 ± 10.8, n = 302) and lower than depressed people (47.2 ± 10.9, n = 90) and high-stress mothers (23.4 ± 1.4, n = 76) [5]. There were 606 (33.6%) IMWs who were considered to have depression symptoms, and 524 (29.0%) IMWs were considered to have anxiety symptoms. A meta-analysis that included 44,365 IMWs reported that the overall prevalence of depression and anxiety among IMWs was 38.99% and 27.31%, respectively [61]. These were similar to the results of this study. The sociodemographic characteristics of group 1 and group 2 are summarized in Table 1. There was no statistically significant difference between the characteristics of the training and validation datasets, which indicated that the random assignment to each subset was successful.

### 3.2. Content Validity

Eight psychologists were invited to assess the degree to which items of the DS are relevant to and representative of defeat. The rating of the experts is displayed in Table 2. The relevance and representativeness of the 13 items on defeat were recognized by all experts, and all items had an I-CVI greater than 0.8. Based on the I-CVI of each item, the Ave-CVI of DS was calculated as 0.977, which was greater than 0.8.

### 3.3. Exploratory Factor Analysis

The value of KMO statistics was 0.943 > 0.6, which indicated that sampling was adequate. Bartlett’s test was significant ($\chi^{2}\;$= 9391.978, *p* < 0.001), which showed the matrix of items was not an identity matrix. The factor analysis was appropriate for the data set.

**Figure 1:**
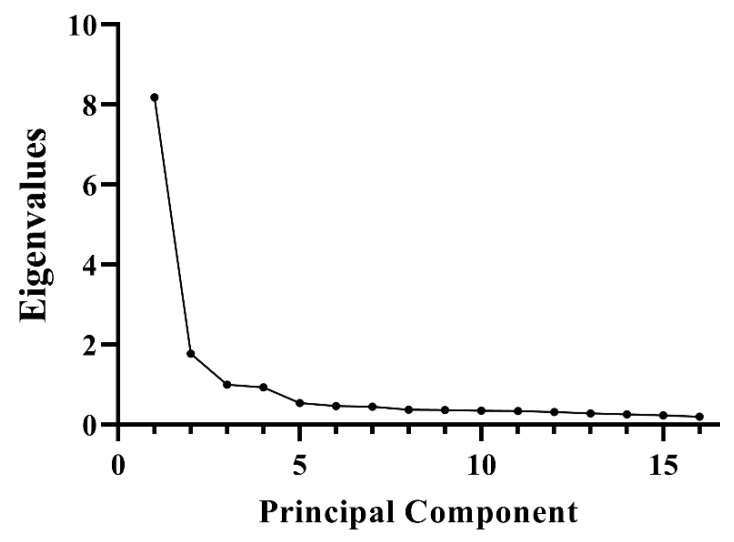
Scree test of DS in IMWs.

According to the scree test (Figure 1), two initial factors were obtained using principal component analysis and orthogonal rotation. Factor 1 contained 13 items, and factor 2 contained the remaining items 2, 4, and 9. The eigenvalues of the two factors were 8.180 and 1.773, respectively, and the cumulative variance contribution rate was 62.208% (Table 3). The factor loading for each item was >0.5 (Table 4).

### 3.4. Confirmatory Factor Analysis

The results of CFA showed the DS should be regarded as a two-factor scale. However, the study of Gilbert et al. indicated that the DS should be a one-factor scale. Therefore, factor loadings and fit indices of one-factor and two-factor models in CFA were compared to determine the dimensionality of the DS. The results of factor loadings indicated that the two-factor model performed better than the one-factor model (Table 5). A comparison of fit indices revealed that the two-factor model was acceptable while the one-factor model was not (Table 6).

### 3.5. Reliability

The Cronbach’s alpha of all 16 items was 0.924, while the Guttman split-half coefficient was 0.88. The Cronbach’s alpha of the two factors was 0.946 and 0.691, respectively. The DS had acceptable internal consistency reliability among IMWs.

### 3.6. Convergent Validity and Discriminant Validity

Based on the factor loadings derived from the CFA, the AVE and CR of the two factors could be calculated. The AVE = 0.5744, CR = 0.945 in factor 1 and AVE = 0.625, CR = 0.830 in factor 2. The correlation coefficient of the two factors was 0.22. These results suggested that the DS had convergent validity and discriminant validity among IMWs.

### 3.7. Logistic Regression

One-factor logistic regression was performed to explore the effects of DS scores on anxiety and depression among the IMWs. For depression, β = 0.127, *p <* 0.0001, and for anxiety, β = 0.131, *p <* 0.0001. The specific results are shown in Table 7.

## 4. Discussion

The results of the content validity test revealed that the information captured by the DS is germane to and reflects defeat. This demonstrates that the DS is a suitable instrument to be introduced to IMWs to quantify defeat. The Kaiser–Meyer–Olkin and Bartlett’s Test of Sphericity suggested that the data collected in this study was amenable to factor analysis. The extraction method of EFA employed in this study was the principal component analysis. After extraction and rotation, two distinct components with eigenvalues greater than 1 were identified. The first component accounted for 51.126% of the total variance, with an eigenvalue of 8.180, while the second component explained 11.082% of the variance, with an eigenvalue of 1.773. The two components thus explained 62.208% of the common variance. The proportion of the total variance explained by the retained factors exceeding 50% was considered reasonable [48]. These results suggested that the DS could extract no more than two factors among IMWs.

The scree test plot was utilized to identify the optimal number of factors. As shown in Figure 1, eigenvalues were represented on the Y-axis, and the 16 component numbers were displayed on the X-axis. The factors were initially extracted with high eigenvalues, followed by smaller factors. The scree test plot showed that the two factors with eigenvalues greater than 1 accounted for a majority of the total variability, while the other factors accounted for a negligible proportion and were deemed to be of lesser significance. Consequently, the two-factor model is deemed to be the optimal choice among the IMWs.

However, Gilbert’s study suggested that the DS should be a one-factor scale [5]. We performed CFA for one-factor and two-factor models, respectively, to compare their fitting. The results indicated that the factor loadings of three reverse items (item 2, item 4, and item 9) in the one-factor model were below 0.4, suggesting that the three reverse items are not representative of the one-factor model [48]. In contrast, they performed well in factor 2 of the two-factor model. The fit of the two models was also noticeably different, as the GFI, RFI, TLI, IFI, and RMSEA indices of the one-factor model failed to reach acceptable levels, whereas they all fit well in the two-factor model. It is important to note that the CMIN/DF value may be unstable due to the sample size, which is a disadvantage when using this index to evaluate the goodness of fit. In this study, 1805 IMWs were included, far surpassing the minimum sample size requirement of 160. This may result in significantly high CMIN, leading to a higher CMIN/DF [62,63]. However, in this study, the CMIN/DF values for both the one-factor and two-factor models in the CFA were comparable due to the same sample size. Despite the fact that the CMIN/DF values for both models were greater than 5, the results of the two-factor model were closer to 5. Therefore, the two-factor model should be the superior option among IMWs.

Factor 1 was labeled as “Decadence” and comprised 13 items that described various forms of failure, such as in competition (e.g., item 15), low social status (e.g., item 10), loss of motivation (e.g., item 16), and loss of confidence (e.g., item 8). Factor 2, consisting of three items, described individuals who were successful (item 2 and item 4) and powerful (item 9). However, these items were scored in reverse, leading to the labeling of Factor 2 as “Low Achievement”.

The results of AVE and CR indicated that the two factors of the DS demonstrated convergent validity among IMWs. The 13 items of Factor 1 (Decadence) were highly similar and contributed to the measurement of decadence, while the three items of Factor 2 (Low Achievement) were also highly similar and contributed to the measurement of low achievement. The AVE of Factor 1 (Decadence) and Factor 2 (Low Achievement) were higher than their correlation coefficients, indicating the presence of discriminant validity among IMWs. This implies that the measure of decadence, as captured by Factor 1, is distinct from the measure of low achievement, as captured by Factor 2.

The regression results showed a positive association between the DS score (as a covariate) and both depression and anxiety (as dependent variables). The beta coefficient (β) was 0.127, and the odds ratio (OR) was 1.136 (95% CI: 1.120, 1.152) when depression was the dependent variable, and β = 0.131 and OR = 1.140 (95% CI: 1.124, 1.156) when anxiety was the dependent variable. These results suggest that an increase in defeat (as measured by the DS score) is associated with an increase in both depression and anxiety among IMWs. Specifically, for every 1 unit increase in the DS score, the probability of suffering from depression increases by 0.136, and the probability of suffering from anxiety increases by 0.140. This highlights the potential importance of defeat in the mental health of IMWs.

IMWs are also at risk for a variety of mental health problems and suicidal ideation or behavior [63,64,65,66,67]. In addition, defeat is also associated with post-traumatic stress disorder and suicide [64,65,66,67]. The introduction of DS may facilitate the study of defeat and other mental health in IMWs.

## 5. Limitations

Several limitations of the present study should be acknowledged. Firstly, the recruitment was conducted using a stratified multi-stage sampling method, and as a result, all internal migrants within a workshop were included, which may result in homogeneity among participants and restrict the generalizability of the findings. Secondly, the measures were self-reported via questionnaires, which raises concerns regarding the subjectivity of the results and the potential for bias. Additionally, information regarding the occupation of the internal migrants was not collected, which would have provided valuable information. Finally, the cross-sectional nature of the study calls for further examination of the predictive relationship between DS scores and depression through longitudinal studies.

## 6. Conclusions

The “Healthy China Action Plan” (2019–2030) highlights the importance of promoting mental health in order to promote social stability and harmonious interpersonal relationships and enhance the public’s sense of well-being. The mental health of IMWs is a highly relevant issue. Research has consistently shown that this population is at a higher risk for various mental health problems, including suicidal thoughts or behaviors. Additionally, experiences of failure and trauma are associated with post-traumatic stress disorder and suicide [64,65,66,67]. These findings underscore the importance of further research into the mental health of IMWs. Our study found that the two-factor model’s DS is an effective and reliable measure of defeat among IMWs in China and also found a positive correlation between defeat and anxiety and depression, indicating that defeat may play a significant role in the mental health of IMWs in China. Consequently, the DS may aid in providing a more comprehensive evaluation of the psychological state of IMWs and furnish information for the development of targeted intervention measures.

## Figures and Tables

**Table 1:** Characteristics of the participants in different groups.

	All Participants n = 1805	Group 1 n = 856	Group 2 n = 949	*p*
Age-Median (IQR)	32 (27.0, 37.0)	32 (27, 36)	32.0 (27, 37)	0.726
Gender				0.978
Male	1214 (67.3%)	576 (67.3%)	638 (67.2%)	
Female	591 (32.7%)	280 (32.7%)	311 (32.8%)	
Nation				0.256
Han nationality	1524 (84.4%)	714 (83.4%)	810 (85.4%)	
Ethnic minority	281 (15.6%)	142 (16.6%)	139 (14.6%)	
Education				0.198
Primary School and below	64 (3.5%)	30 (3.5%)	34 (3.6%)	
Junior High School	995 (55.1%)	493 (57.6%)	502 (52.9%)	
High School	585 (32.4%)	259 (30.3%)	326 (34.4%)	
junior college	94 (5.2%)	43 (5.0%)	51 (5.4%)	
Undergraduate	19 (1.1%)	12 (1.4%)	7 (0.7%)	
Other	48 (2.7%)	19 (2.2%)	29 (3.1%)	
Marriage				0.759
Unmarried with no boyfriend/girlfriend	555 (30.7%)	260 (30.4%)	295 (31.1%)	
Unmarried but with a boyfriend/girlfriend	164 (9.1%)	88 (10.3%)	76 (8.0%)	
Married	967 (53.6%)	441 (51.5%)	526 (55.4%)	
Divorce/widowhood	47 (2.6%)	24 (2.8%)	23 (2.4%)	
Others	72 (4.0%)	43 (5.0%)	29 (3.1%)	
Wage ($) *				0.201
Unknown	56 (3.1%)	17 (2.0%)	39 (4.1%)	
Less than 154.67	25 (1.4%)	13 (1.5%)	12 (1.3%)	
154.67–463.85	141 (7.8%)	49 (5.7%)	92 (9.7%)	
464.00–773.18	1099 (60.9%)	552 (64.5%)	547 (57.6%)	
773.34–1082.51	447 (24.8%)	214 (25.0%)	233 (24.6%)	
1082.67–1546.52	29 (1.6%)	8 (0.9%)	21 (2.2%)	
Over 1546.67	8 (0.4%)	3 (0.4%)	5 (0.5%)	

Data are presented as n (%) or median (IQR). * Exchange rate reference time: 5 August 2021.

**Table 2:** Ratings on 16 items of DS by 8 experts.

Items	Expert 1	Expert 2	Expert 3	Expert 4	Expert 5	Expert 6	Expert 7	Expert 8	Number of Agreements	Item-CVI1
1	4	4	4	4	4	4	3	4	8	1.000
2	3	4	3	3	2	4	3	4	7	0.875
3	4	3	4	3	3	4	4	4	8	1.000
4	4	3	3	3	3	4	4	3	8	1.000
5	4	4	4	4	4	3	4	3	8	1.000
6	4	3	4	3	4	4	4	3	8	1.000
7	3	3	3	4	1	3	4	4	7	0.875
8	4	3	4	3	3	4	4	4	8	1.000
9	4	3	3	3	4	3	3	4	8	1.000
10	4	4	3	4	3	4	4	4	8	1.000
11	3	3	2	3	4	4	3	4	7	0.875
12	4	4	4	4	4	4	4	4	8	1.000
13	4	3	3	4	4	4	4	4	8	1.000
14	4	4	4	4	3	4	4	3	8	1.000
15	4	4	3	3	4	3	4	3	8	1.000
16	4	4	4	3	3	4	4	4	8	1.000

**Table 3:** Eigenvalues and total variance explained (n = 856).

Component	Initial Eigenvalues			Extraction Sums of Squared Loadings			Rotation Sums of Squared Loadings		
	Total	% of Variance	Cum%	Total	% of Variance	Cum%	Total	% of Variance	Cum%
1	8.180	51.126	51.126	8.180	51.126	51.126	7.882	49.263	49.263
2	1.773	11.082	62.208	1.773	11.082	62.208	2.071	12.944	62.208
3	0.994	6.213	68.421						
4	0.928	5.802	74.222						
5	0.535	3.341	77.564						
6	0.466	2.915	80.479						
7	0.444	2.776	83.256						
8	0.374	2.338	85.593						
9	0.360	2.250	87.843						
10	0.342	2.136	89.980						
11	0.336	2.099	92.079						
12	0.313	1.954	94.033						
13	0.279	1.742	95.775						
14	0.257	1.608	97.383						
15	0.227	1.418	98.801						
16	0.192	1.199	100.000						

Extraction Method: Principal Component Analysis. Cum means Cumulative.

**Table 4:** Item factor loadings of the two-factor model (n = 856) in EFA.

		Two-Factor Model	
Item		Factor 1	Factor 2
item1	I feel that I have not made it in life	0.630	
item2	I feel that I am a successful person (R)		0.865
item3	I feel defeated by life	0.726	
item4	I feel that I am basically a winner (R)		0.881
item5	I feel that I have lost my standing in the world	0.664	
item6	I feel that life has treated me like a punchbag	0.698	
item7	I feel powerless	0.807	
item8	I feel that my confidence has been knocked out of me	0.753	
item9	I feel able to deal with whatever life throws at me (R)		0.593
item10	I feel that I have sunk to the bottom of the ladder	0.773	
item11	I feel completely knocked out of action	0.805	
item12	I feel that I am one of life’s losers	0.863	
item13	I feel that I have given up	0.819	
item14	I feel down and out	0.857	
item15	I feel I have lost important battles in life	0.832	
item16	I feel that there is no fight left in me	0.814	

Note: (R) means reverse coded item.

**Table 5:** Item factor loadings of the one-factor model and two-factor model (n = 949) in CFA.

		One-Factor Model	Two-Factor Model	
Item			Factor 1	Factor 2
item1	I feel that I have not made it in life	0.587	0.586	
item2	I feel that I am a successful person (R)	0.137		0.735
item3	I feel defeated by life	0.664	0.664	
item4	I feel that I am basically a winner (R)	0.148		0.762
item5	I feel that I have lost my standing in the world	0.670	0.670	
item6	I feel that life has treated me like a punchbag	0.650	0.650	
item7	I feel powerless	0.789	0.788	
item8	I feel that my confidence has been knocked out of me	0.692	0.691	
item9	I feel able to deal with whatever life throws at me (R)	0.237		0.434
item10	I feel that I have sunk to the bottom of the ladder	0.747	0.733	
item11	I feel completely knocked out of action	0.788	0.788	
item12	I feel that I am one of life’s losers	0.837	0.838	
item13	I feel that I have given up	0.827	0.827	
item14	I feel down and out	0.880	0.880	
item15	I feel I have lost important battles in life	0.845	0.845	
item16	I feel that there is no fight left in me	0.824	0.825	

Note: (R) means reverse coded item.

**Table 6:** Model fit indices of the one-factor and two-factor model (n = 949).

Index	Acceptable Value	One-Factor Model	Two-Factor Model
CMIN/DF	<5	9.751	5.343
GFI	>0.9	0.883	0.932
CFI	>0.9	0.910	0.956
NFI	>0.9	0.901	0.946
RFI	>0.9	0.881	0.935
IFI	>0.9	0.910	0.956
TLI	>0.9	0.892	0.946
sRMR	<0.08	0.0695	0.0522
RMSEA	<0.08	0.096	0.068

Note: DF = degree of freedom, CMIN = chi-square fit statistics.

**Table 7:** Results of one-factor logistic regression.

Dependent Variables	Covariates	β	S.E.	Wald	df	*p*	OR (95%CI)
Depression	DS score	0.127	0.007	315.117	1	<0.001	1.136 (1.120, 1.152)
Anxiety	DS score	0.131	0.007	318.743	1	<0.001	1.140 (1.124, 1.156)

## Data Availability

Data are available on request due to privacy and ethical restrictions. The data presented in this study are available on request from the corresponding author. The data are not publicly available due to protection of participants’ privacy.
